# Post-extubation Stridor in a Case of Intracranial Bleed: Assessing Airway Patency Prior to Extubation Using Cuff Leak Test

**DOI:** 10.7759/cureus.33632

**Published:** 2023-01-11

**Authors:** Pradnya Diggikar, Simranbir Bhullar, Prashant Gopal

**Affiliations:** 1 General Medicine, Dr. D. Y. Patil Medical College, Pimpri-Chinchwad, IND

**Keywords:** intracranial bleed, corticosteroids, stridor, laryngeal edema, endotracheal intubation

## Abstract

Laryngeal edema is a common complication of endotracheal intubation. It may range from mild and asymptomatic to respiratory distress and severe stridor leading to subsequent reintubation. It is crucial to assess the patency of the airway before extubation to identify patients with a risk of developing laryngeal edema. To prevent post-extubation laryngeal edema (PLE), intravenous corticosteroids or nebulized corticosteroids appear to be reasonably effective, reducing the need for reintubation by more than half. We present a case of a 59-year-old male who presented with an intracranial bleed and aspiration pneumonia. The patient developed PLE and was reintubated due to respiratory distress and treated with intravenous and nebulized corticosteroids. The patient was extubated two days later after adequate cuff leak test (CLT) results. If PLE causes respiratory distress, reintubation is the only definitive treatment and should not be delayed.

## Introduction

Endotracheal intubation is a lifesaving procedure frequently complicated by laryngeal edema (LE) caused by trauma to the larynx. It may range from mild and asymptomatic to respiratory distress and severe stridor leading to subsequent reintubation. It may result in a reduction of the airway lumen with respiratory distress immediately following extubation. Narrowing of the airway lumen results in increased airflow velocity, resulting in post-extubation stridor (PES). Significant risk factors include prolonged duration of intubation, the use of a large-sized endotracheal (ET) tube with high cuff pressures, female gender, and traumatic intubation. Although the precise relationship between clinical symptoms and airway lumen narrowing is unknown, the occurrence of PES and respiratory distress is assumed to indicate narrowing by more than 50% [1].

Prior to extubation, it is necessary to evaluate the patency of the airway to determine patients at risk of laryngeal edema. Since Miller (&) Cole examined 100 mechanically ventilated patients in 1996 [2], the cuff leak test (CLT) has been frequently employed due to its non-invasive nature and lack of the requirement for sophisticated equipment [3]. Due to the possibility of recurrence of airway lumen narrowing at the anastomosis site and the fact that these patients are frequently at a higher surgical risk, surgical resection for post-intubation tracheal stenosis management continues to be a contentious subject. Stent insertion, laser resection, and balloon bronchoplasty are alternate treatment options [4].

## Case presentation

A 59-year-old male with a known case of hypertension presented with left-sided hemiplegia, global aphasia, and respiratory distress for the past day. He was not on any hypertensive or antiplatelet medication and there was no history of any previous cerebrovascular accident or ischemic heart disease. The patient had tachycardia (130bpm), tachypnea (RR-38/min), and blood pressure was 190/110 mmHg. He was rated 6/15 (E1V1M4) on the Glasgow coma scale (GCS) and oxygen saturation at room air was 82%. The patient was intubated in view of low GCS and respiratory distress. An urgent non-contrast computerized tomography (NCCT) brain showed an acute intraparenchymal hemorrhage in the region of the right putamen. High-resolution computerized tomography (HRCT) thorax revealed bilateral non-homogenous opacities (right>left) suggestive of aspiration pneumonia. The patient's laboratory investigations are listed in Table 1.

**Table 1:** Laboratory investigations of the patient on admission

Test name	Test value	Normal range
Hemoglobin	11.2 g/dL	14.0 – 17.5 g/dL
Total leukocyte count (TLC)	16,700/mm^3^	4000-10,000/mm^3^
Platelets	2,34,000/mm^3^	1,50,000 - 4,50,000/mm^3^
Procalcitonin	4.8 ng/ml	<0.05 ng/mL
Urea	134 mg/dL	17 – 49 mg/dL
Creatinine	2.8 mg/dL	0.6 – 1.2 mg/dL
C-reactive protein (CRP)	156 mg/L	< 2 mg/L

Liver function tests were normal. Ultrasonography abdomen showed no abnormality and 2D echocardiography revealed moderate concentric left ventricular hypertrophy with a 58% ejection fraction.

The patient was admitted to the intensive care unit (ICU) and started on antibiotics, antihypertensive, and antiedema medication like diuretics. He was on ventilatory support for eight days and then extubated. The patient's GCS pre-extubation was 10T/15 (E4VTM6). The patient was oriented and obeying commands but there was no improvement in power on the left side.

**Figure 1:**
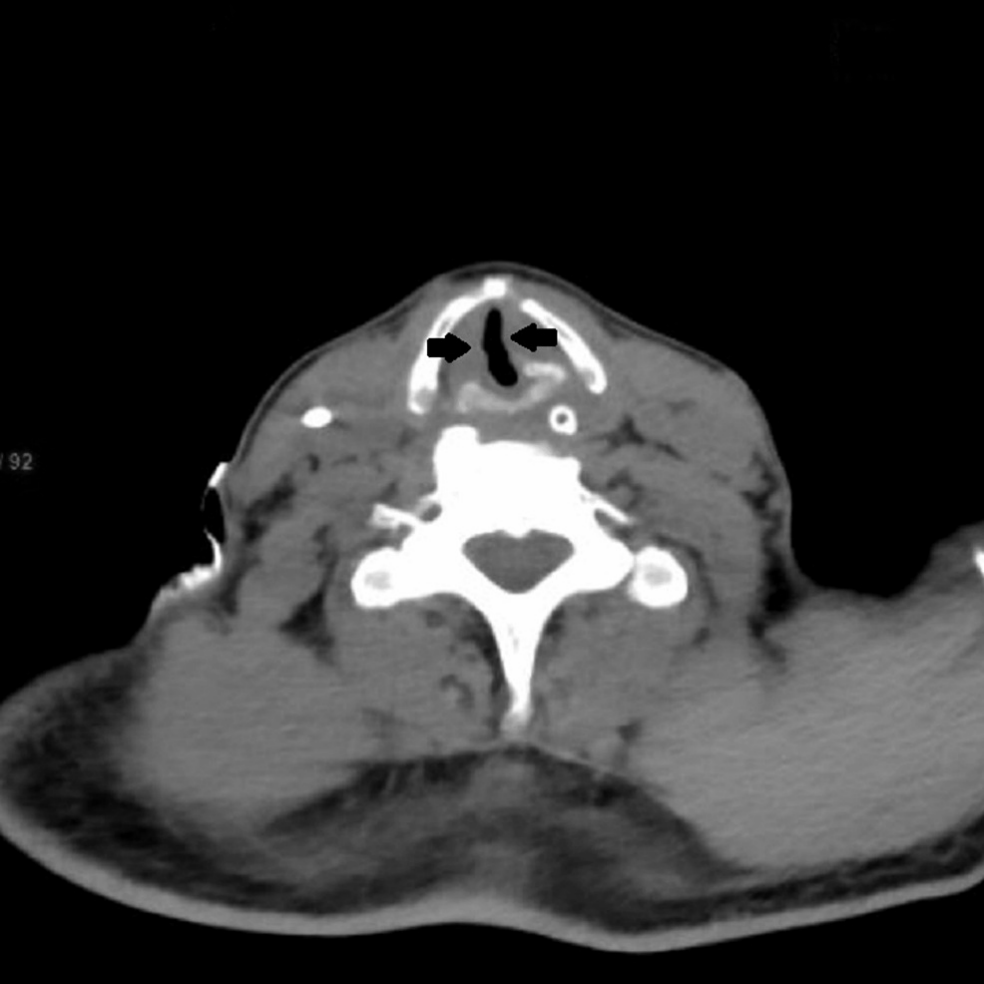
CT of the neck showing soft tissue edema with significant airway compromise

**Figure 2:**
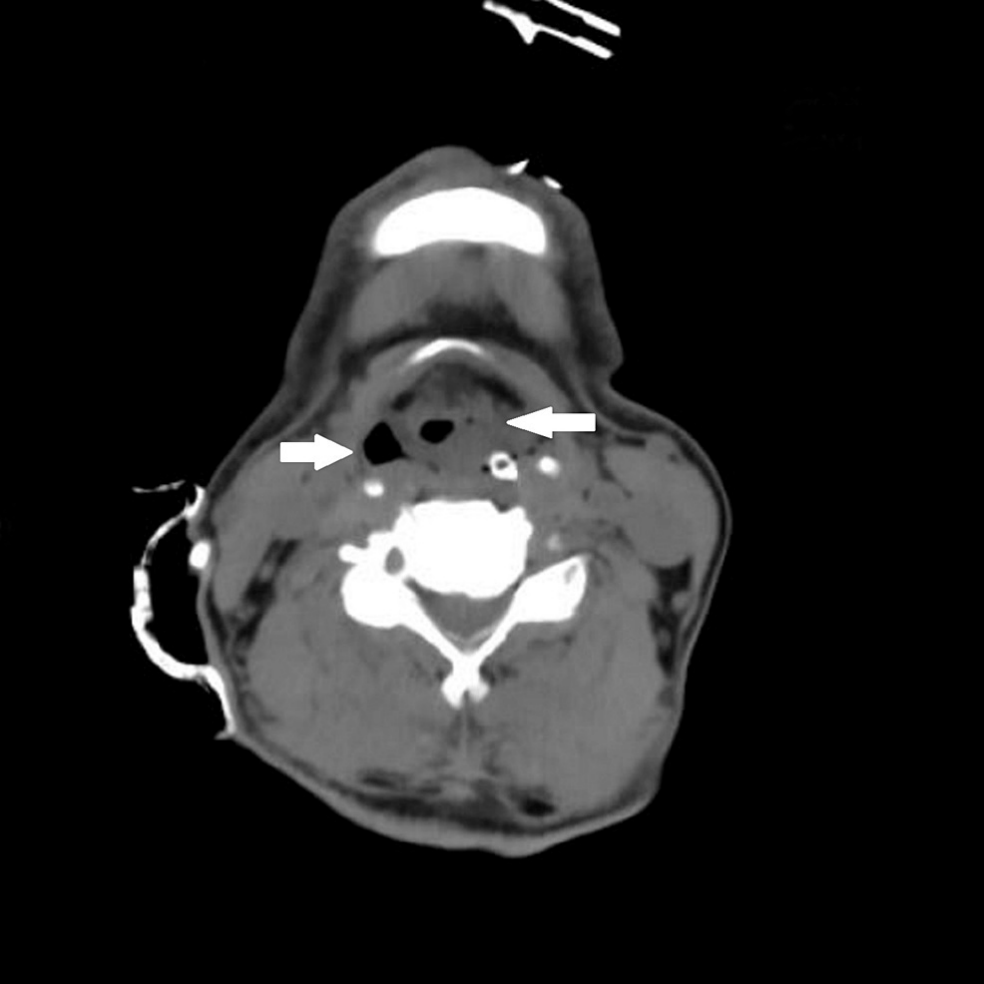
CT of the neck revealing soft tissue edema with significant airway compromise

Four hours post-extubation; the patient developed labored breathing and stridor. There were no new changes in the chest X-ray. Video direct laryngoscopy showed edematous vocal cords. A CT of the neck was done which showed bilaterally enlarged edematous vocal cords and soft tissue edema with thickened left aryepiglottic fold causing obliteration of the left pyriform fossa with significant airway compromise (Figures 1, 2). The patient was reintubated in view of respiratory distress and treated with intravenous (dexamethasone 4mg IV TDS) and nebulized corticosteroids. Cuff leak tests were done regularly with the bulb inflated during inspiration and deflated before expiration; the maneuver was repeated five times during each test and the values averaged. The patient responded to treatment and showed improved oxygenation levels and CLT results (Table 2) and was extubated two days later. After a hospital stay of 24 days, the patient was discharged and is asymptomatic on follow-up.

**Table 2:** Cuff leak test values post reintubation

Time	6 hours	12 hours	24 hours	36 hours	48 hours
Volume (leak)	84ml	96ml	112ml	136ml	158ml

## Discussion

Stenosis following intubation is still a significant acquired cause of tracheal luminal narrowing. When cuff pressures are greater than the capillary pressure of the tracheal mucosa (30mm of Hg), ischemia and edema develop in the mucosa between the underlying cartilages and the balloon cuff. In intensive care units, the use of ET tubes with a large area of contact (high-volume, low-pressure cuff) has significantly decreased the chances of post-intubation tracheal stenosis [4]. In most cases, endotracheal intubation damages the airway, resulting in laryngeal edema, ulcerations, and vocal cord damage. Severe respiratory failure, trauma during intubation, female gender, smaller height-to-tube diameter ratio, longer duration of intubation, infections, longer duration of ICU stay, and low GCS are all risk factors for PLE [1]. The patient was not assessed for laryngeal edema prior to extubation and subsequently developed PLE.

Pre-extubation airway patency assessment using the CLT can assist in identifying patients at risk for PLE and avoiding the need for reintubation. Elimination of potential risk factors may prevent the occurrence of PLE. A suitable endotracheal tube should be chosen. Maximum sizes generally accepted are 8mm for men and 7mm for women. Since patients with PES have a consistently longer duration of intubation compared to patients without PES, the duration of intubation should be kept to a minimum. Frequent monitoring of cuff pressures helps prevent the development of pressure ulcers.

Since endotracheal intubation prevents direct visualization of the upper airway, the CLT is used to detect the presence of laryngeal edema and post-extubation luminal stenosis. The normal practice involves deflating the cuff during volume control ventilation (with a tidal volume of 10 ml/kg) and measuring the expired tidal volume a few breaths later. The difference between the expiratory tidal volume with and without a deflated cuff is used to determine the leak [5]. Deflating the cuff of an ET tube causes air leaks around the tube when there is no edema. A failed CLT, on the other hand, indicates that there is little or no air leakage, indicating the presence of laryngeal edema.

The American Thoracic Society and American College of Chest Physicians published a clinical practice guideline in 2017 recommending using CLTs to screen for PLE in intubated patients who meet the criteria for extubation. The CLT has a high specificity but a low sensitivity for PLE according to several studies. Thus, it is better at ruling in than ruling out potential PLE. The CLT has several limitations, including susceptibility to the tube-to-laryngeal luminal diameter relationship, respiratory system compliance and resistance, inspiratory and expiratory flow and time, and airway collapse. Additionally, coughing during cuff deflation impairs the accuracy of leak volume measurement and reduces reproducibility [6].

Laryngeal or upper airway mucosal edema is a frequent cause of airway occlusion following extubation and is believed to result from direct mechanical trauma to the larynx caused by the endotracheal tube [7]. Reintubation should be undertaken as early as possible if there is respiratory distress owing to PLE. While non-invasive ventilation (NIV) is effective in preventing intubation in general, it has been ineffective in treating respiratory insufficiency following extubation. Nebulized epinephrine and intravenous corticosteroids are currently recommended for the management of PLE. Corticosteroids reduce PLE by inhibiting both the dilation and permeability of capillary vessels and the inflammatory response. However, due to the lack of research on the efficacy of corticosteroids in treating PLE, there is no evidence regarding the optimal dose. Methylprednisolone 20mg to 40mg or dexamethasone 5mg may be recommended, with therapy continuing for 24 to 48 hours post-extubation, based on the dosages currently being used to prevent PLE [1].

## Conclusions

Post-extubation laryngeal edema is a common intubation complication that results in up to 10% of all extubated patients requiring reintubation. The CLT, despite its limitations, is a useful test to assess airway patency in intubated patients. Pre-treatment with nebulized corticosteroids or intravenous corticosteroids following extubation appears to be fairly effective at preventing PLE, significantly reducing the need for reintubation. However, the absence of reliable indicators makes identifying individuals at high risk of PLE difficult. If PLE is present, a combination of corticosteroids and nebulized epinephrine is recommended. However, if PLE results in respiratory insufficiency, the only definitive treatment is reintubation, which should not be delayed.
